# Contextual inequalities in specialized dental public health care in Brazil

**DOI:** 10.1590/1807-3107bor-2024.vol38.0023

**Published:** 2024-04-05

**Authors:** Ana Luiza Cardoso PIRES, Francine dos Santos COSTA, Otávio Pereira D’ÁVILA, Rodrigo Varella de CARVALHO, Marcus Cristian Muniz CONDE, Marcos Britto CORREA, Flávio Fernando DEMARCO, Luiz Alexandre CHISINI

**Affiliations:** (a) Universidade Federal de Pelotas – UFPel, School of Dentistry, Department of Restorative Dentistry, Pelotas, RS, Brazil.; (b) Universidade Federal de Pelotas – UFPel, School of Dentistry, Pelotas, RS, Brazil.; (c) Universidade Federal de Pelotas – UFPel, School of Dentistry, Department of Social Dentistry, Pelotas, RS, Brazil.; (d) Universidade Federal de Juiz de Fora – UFJF, Department of Restorative Dentristry, Juiz de Fora, MG, Brazil.; (e) Universidade do Vale do Taquari–Univates, Center for Biological Sciences and Health, Department of Restorative Dentistry, Lajeado, RS, Brazil.

**Keywords:** Public Health, National Health Programs, Socioeconomic Factors, Dentistry

## Abstract

The present study aimed to investigate the contextual inequalities of specialized public dental care (SPDC) in Brazil. The outcome was the trajectory of dental specialized production in municipalities with SPDC (from 2015 to 2017) obtained by group-based trajectory modeling. A Poisson regression model was used to analyze the factors associated with the high trajectory of SPDC production. The inequality indicators for SPDC production were the slope index and the concentration index according to contextual factors. The study included 954 SPDC units distributed across 893 municipalities. Among the municipalities evaluated, 62.9% had a low trajectory of SPDC. Large-sized municipalities had the highest production (IRR = 2.84, 95%CI: 1.94–4.14) and the southern region had the lowest production (IRR = 0.73, 95%CI: 0.58–0.92). Municipalities presenting a very high human development index (HDI) showed the greatest SPDC production (IRR = 3.34, 95%CI: 1.09–10.24), as well as municipalities with the highest tertile of schooling rate (IRR = 1.23, 95%CI: 1.00–1.50). The absolute inequality was 52.1 percentage points for the average monthly wage (p < 0.001), 61.0 percentage points for the HDI (p < 0.001), -22.1 for infant mortality rate (p <0.001), and 14.8 for the schooling rate (p = 0.012). Thus, there are contextual inequalities in the Brazilian SPDC. Higher scores for social indicators were associated with better SPDC performance.

## Introduction

The Brazilian society is widely marked by social^1^ and racial^2,3^ inequalities that affect the population’s quality of life.^4^ Such inequalities can affect the National Public Health System, Sistema Único de Saúde (SUS), which is the largest public health system in the world and is based on the principle that every citizen should have the right to health and that the federal government should provide universal access to health care. In Brazil, nearly 75% of the population has access to health care.^5,6^ In dental care, health inequalities have always been historically observed.^5^ The Family Health Strategy was created to rearrange the Primary Health Care (PHC) model in Brazil, including dentistry in the program from the 2000s onwards^7^ through the National Oral Health Policy or “Smiling Brazil Program” (*Programa Brasil Sorridente*, in Portuguese).^5^

The strong expansion of oral health services in PHC was complemented by structuring a network of specialized public dental care (SPDC), boosting the Smiling Brazil Program, to offer comprehensive oral health care via the National Public Health System.^5^ Thus, since 2006, access to the secondary care network through Dental Specialties Centers (DSC) has been expanded and implemented throughout the Brazilian territory.^8,9^ DSCs are integrated into a complex care network, providing moderately complex procedures.^5^

The DSC implementations provided peripheral populations with wide coverage and access to SPDC.^10,11^ However, there are persistent problems in oral health due to the reduced access and use of dental services, mainly in the poorest population,^12^ causing further inequalities in oral health services. The relationship between a population’s socioeconomic background and health status is well established in the literature: the social gradient in health and disease follows the socioeconomic level of the population, and the lower the position occupied in the social hierarchy, the worse the individuals’ health status.^13^

Therefore, strategies for progressively expanding access to SPDC and directing public resources to programmatic targets are known to be associated with the reduction of health inequalities.^10^ Besides, advances in epidemiological indicators have been observed as a result of the National Oral Health Policy actions.^5^ However, these actions alone are not sufficient to solve oral health inequalities. Most of these inequalities arise from the different contexts that underlie the social determinants of health.^13-16^ Therefore, actions involving ethnic-racial, behavioral, gender heterogeneity, and risk behavior issues need to be integrated into the existing ones.^17^ National assessments of the factors influencing the delivery of public dental care by SUS are based mainly on primary care, with little attention to the analysis of inequalities.^7,11,18^ Thus, the objective of the present study was to investigate contextual inequalities of SPDC in Brazil.

## Methodology

The present study was reported following the STROBE guidelines for observational studies.

### Study design

This is a retrospective longitudinal ecological study using secondary data from the SUS database (DATASUS; http://www.datasus.gov.br), from the National Register of Health Establishments (CNES; http://cnes.datasus.gov.br/), and the Brazilian Institute of Geography and Statistics (IBGE; http://www.ibge.gov.br). All data were extracted from open-access secondary databases in the public domain, eliminating the need for approval by the Research Ethics Committee. SPDC registered in the CNES and active throughout the period from January 2015 to December 2017 were included in the present study. The data were collected during the period from January to October 2020.

### Independent variables

The independent variables assessed in this study were population size of municipalities, the Human Development Index (HDI), coverage of PHC and PHC with the oral health team, average monthly income of the population, schooling rate (for ages 6 to 14 years), and infant mortality rate (IBGE; https://cidades.ibge.gov.br/). The absolute estimated population of each municipality was obtained through IBGE data, considering the projections for the year 2016 and categorized as a) ≤ 20,000; b) 20,001 to 50,000; c) 50,001 to 150,000; and d) >150,000.^19,20^

The HDI is a comparative measure that summarizes achievements in three fundamental dimensions: long and healthy life, access to knowledge, and a good standard of living. The score is obtained by the geometric mean of the standardized indices for each of the dimensions. Thus, the HDI of each municipality was collected from data provided by IBGE and categorized as a) low, HDI less than 0.550; b) average, between 0.550 and 0.699; c) high, between 0.700 and 0.799; and d) very high, equal to or greater than 0.800.

The PHC coverage data were obtained from the Primary Care Information System (SIAB, Sistema de Informações da Atenção Básica, in Portuguese) for the year 2016. It was categorized as a) absent (coverage equal to 0%); b) incipient (population coverage other than zero and less than 30%; c) intermediate (between 30% and 70% or ≥ 70% and less than four years after implementation); and d) consolidated (≥ 70% and with at least four years of implementation).^21^ PHC coverage with an oral health team was obtained from the SIAB for the year 2016 and categorized as a) absent (coverage equal to 0%); b) incipient (population coverage other than zero and less than 30%; c) intermediate (between 30% and 70% or ≥ 70%); and d) consolidated (≥ 70%).

Schooling rates for ages 6 to 14 years and the average local wage in minimum wages were collected from the IBGE data, considering the 2010 Brazilian census and categorized in tertiles.

### Dependent variable

This study examined the trajectory of dental specialized services in municipalities with SPDC. First, we mapped Brazilian municipalities with SPDC, which had to be registered and active during the evaluated period (2015 to 2017) in the National Register of Health Establishments. In addition, we collected the number and type (I, II, or III) of DSCs in each municipality. The main differences between the services lie in the number of professionals and the number of procedures offered by each center. The Brazilian Ministry of Health provides three types of incentives for the implementation and operation of DSC. The incentive for implementation is 60,000 Brazilian Reais (R$) for DSC Type I (with three dental chairs), R$ 75,000 for DSC Type II (with four to six chairs), and R$ 120,000 for DSC Type III (with more than seven dental chairs). The monthly operational incentive ranges according to DSC Type: R$ 8,250 for DSC Type I, R$ 11,000 for DSC Type II, and R$ 19,250 for DSC Type III. Therefore, the DSCs must maintain a minimum monthly production in each specialty, and failure to meet such production for two consecutive months or three non-consecutive months within one year will lead to the suspension of operational incentives. After mapping, the assortment of specialized dental procedures (outlined in Interministerial Ordinance no. 1464, which establishes the financing of DSCs) was investigated in the SUS Outpatient Information System (SIA/SUS).

The procedures were gathered into four groups as outlined in the Interministerial Ordinance: a) basic procedures for patients with special needs; b) specialized procedures for periodontic treatment; c) specialized procedures for minor oral surgery; and d) specialized procedures for endodontic treatment. Each category was then dichotomized as to the achievement of minimal production following the specifications of the Interministerial Ordinance 1.464/2011.^22^ The production analysis was made by the annual number of procedures performed by each municipality with DSC, according to the number of DSCs in each municipality and the type of each DSC. Thus, for each procedure group, the municipality could a) achieve or b) not achieve the minimal production. Therefore, an annual production score was created for each group. Five production achievement scores were considered for each year, with a) score 0 for municipalities that did not meet any goals; b) score 1 for municipalities that met one goal; c) score 2 for municipalities that met two goals; d) score 3 for municipalities that met three goals; and e) score 4 for municipalities that met all goals.

Group-based trajectory modeling was used to identify different trajectories of production over the years.^2,23^ The method was designed to identify groups of municipalities sharing similar trajectories of production over time. The models were estimated using the “traj” command in the Stata 16.0 software package. Three time periods (2015, 2016, and 2017) and five production scores were used in the analysis.

**Figure 1:**
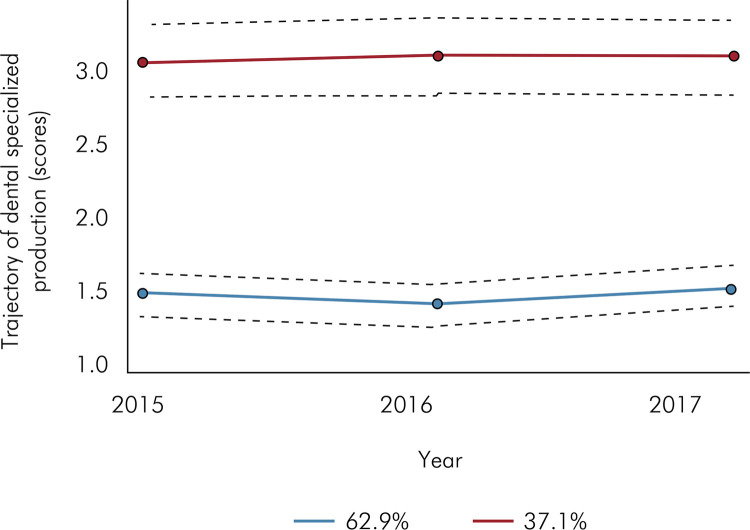
Trajectory of dental specialized production in Brazil from 2015 to 2017 (group-based trajectory modeling) (n=839 municipalities)

The parameters for the trajectory model were determined based on the maximum probability of the quasi-Newton method. The model selection method considers the estimation of the latent number of categories and the order of the polynomial for each latent trajectory. The final number of trajectories was established when the sequential comparisons of the Bayesian information criterion (BIC) and the adjusted BIC between the model with k and k + 1 trajectories did not result in any further substantial difference in the value of the BIC, i.e., no better adjustment according to the BIC.^24,25^ For each group, quadratic trajectories were considered, starting with only one group in a null model. However, four models were initially identified and the two-class model was considered to be the best-fit model, as it had the lowest BIC (1856.45) and the lowest adjusted BIC per sample (1852.61) (Figure 1).

Thus, we observed a group of municipalities with high production and another one with low dental production. The details of the trajectory constructions are presented in Table 1.

**Table 1:** Goodness-of-fit statistics and parameter estimates for the trajectory of dental specialized production. (n = 893 municipalities).

Groups	Intercept	Standard error	p-value	Linear slope	Standard error	p-value	Quadratic slope	Standard error	p-value	AIC	BIC	Sample adjusted BIC
1	0.73	0.03	< 0.01	-0.01	0.08	0.87	0.00	0.04	0.93	1867.58	1875.20	1873.56
2	0.03	0.07	< 0.01	-0.04	0.14	0.75	0.01	0.07	0.85	1838.65	1856.45	1852.61
	1.05	0.05	< 0.01	0.00	0.11	0.96	-0.00	0.05	0.96			
3	0.37	0.11	0.01	-0.04	0.20	0.82	0.01	0.09	0.89	1842.65	1870.62	1964.58
	0.37	0.11	0.01	-0.04	0.22	0.83	0.01	0.10	0.90			
	1.05	0.05	< 0.01	0.00	0.11	0.96	-0.00	0.05	0.96			
4	0.37	0.12	0.01	-0.04	0.22	0.83	0.01	0.10	0.90	1.846.65	1884.79	1876.55
	0.37	0.10	0.01	-0.04	0.20	0.82	0.01	0.09	0.89			
	1.05	0.09	< 0.01	0.00	0.17	0.97	-0.00	0.08	0.97			
	1.05	0.08	<0.01	0.00	0.15	0.97	-0.00	0.07	0.97			

AIC: Akaike information cCriterion; BIC: Bayesian information criterion. *Significance p < 0.05

### Statistical analysis

The STATA software package, version 16 (https://www.stata.com), was used to analyze the data. First, a descriptive analysis was performed to determine the relative and absolute frequency of the independent variables and the trajectory of dental specialized production using Fisher’s exact test.

To analyze the factors associated with the high trajectory of dental specialized production, a Poisson regression model was used. Variables with p < 0.250 in the unadjusted model were not included in the final model. A backward stepwise procedure was used to select the variables that should be kept in the final model. Only variables with p ≤ 0.05 were maintained in the final model. The incidence rate ratio (IRR) and their 95% confidence intervals were estimated. The quality of the model fit was assessed using deviation (-2 log likelihood).

To assess inequalities in the trajectory of dental specialized production, the frequencies according to the average monthly minimum wages, HDI, infant mortality rate, and schooling rate for children aged 6 to 14 years were presented in the equiplot (http://www.equidade.org/equiplot). The inequality indicators used were the slope index and the concentration index for the trajectory of dental specialized production. The slope index indicates absolute inequality (in percentage points) ranging from -100 to + 100%. The positive results demonstrate a higher frequency of the outcome for the largest indicators. Negative results demonstrate a higher frequency of the outcome for the lowest indicators. The concentration index indicates relative inequality, which ranges from -1 to +1, with positive results indicating a greater concentration of DSCs with the high achievement of goals in the municipalities with the highest stratified areas. The latter result is presented in percentages.

## Results

A total of 1,099 DSCs distributed across 906 municipalities were identified, of which 13.1% were closed during the period. The main reasons for their inactivity were retirement and suspension of contracts. Thus, 954 DSCs located in 893 municipalities were included in the study. The Northeast region had the highest number of DSCs (Table 2). Table 3 presents the descriptive analysis of the independent variables and the trajectory of dental specialized production.

**Table 2:** Distribution of Dental Specialty Centers by Brazilian regions. (n = 893 municipalities).

Regions	Dental Specialty Centers		
	Type I	Type II	Type III
	Total (mean)	Total (mean)	Total (mean)
Southeast	132 (29.6)	162 (39.9)	26 (25.7)
North East	212 (47.4)	146 (36.0)	40 (39.7)
Midwest	18 (4.0)	32 (9.8)	9 (8.9)
North	27 (6.0)	20 (4.8)	9 (8.9)
South	58 (13.0)	46 (11.3)	17 (16.8)

**Table 3:** Descriptive analysis of independent variables according to the goal achievement trajectory of DSCs, Brazil (n = 893 municipalities).

Variables	Trajectory of dental specialized production		
	Low n (%)	High n (%)	
Region			< 0.001
Southeast	143 (47.8)	156 (52.2)	
Northeast	297 (78.6)	81 (21.4)	
Midwest	26 (49.1)	27 (50.9)	
North	27 (54.0)	23 (46.0)	
South	68 (60.7)	44 (39.3)	
Population			< 0.001
≤ 20,000	132 (82.5)	28 (17.5)	
20,001–50,000	230 (73.5)	83 (26.5)	
50,001–150,000	159 (58.5)	113 (41.5)	
> 150,000	40 (27.2)	107 (72.8)	
HDI			< 0.001
Low (<,55)	23 (88.5)	3 (11.5)	
Average (0,55–0,699)	317 (78.3)	88 (21.7)	
High (0,70–0,799)	219 (51.6)	205 (48.4)	
Very high (≥.80)	2 (5.4)	35 (94.6)	
Coverage of PHC			< 0.001
Absent	6 (54.5)	5 (45.5)	
Incipient	20 (36.4)	35 (63.6)	
Intermediate	108 (46.1)	126 (53.9)	
Consolidated	427 (72.1)	165 (27.9)	
Coverage of PHC (with oral health team)			< 0.001
Absent	27 (58.7)	19 (41.3)	
Incipient	78 (41.0)	112 (59.0)	
Intermediate	154 (60.6)	100 (39.4)	
Consolidated	302 (75.1)	100 (24.9)	
Average local wage (tertile)			< 0.001
1^st^ (low)	274 (78.1)	77 (21.9)	
2^nd^	180 (64.5)	99 (35.5)	
3^rd^ (high)	107 (40.8)	155 (59.2)	
Schooling rates			0.044
1^st^ (low)	214 (67.7)	102 (32.3)	
2^nd^	179 (62.6)	107 (37.4)	
3^rd^ (high)	168 (57.9)	122 (42.1)	
Infant mortality rate			< 0.001
1^st^ (low)	166 (56.8)	126 (43.1)	
2^nd^	167 (57.8)	122 (42.2)	
3^rd^ (high)	208 (71.7)	82 (28.3)	

Table 4 details the unadjusted and adjusted association of the independent variables with the trajectory of dental specialized production. Considering the adjusted association, we observed that large population sizes (IRR = 2.84, 95%CI: 1.94–4.14), municipalities with a very high HDI (IRR = 3.34, 95%CI: 1.09–10.24), and the highest tertile of schooling rate (IRR = 1.23, 95%CI: 1.00–1.50) were associated with high trajectory of dental specialized production.

**Table 4:** Unadjustedc and adjusteda incidence rate ratio (IRR) between contextual variables and high trajectory of dental specialized production in public services, Brazil. (n = 893 municipalities)

Variables	IRR^u^ (95% CI)	p-value	IRR^a^ (95% CI)	p-value
Region		< 0.001		0.005
Southeast	1		1	
Northeast	0.41 (0.33–0.51)		0.76 (0.56–1.02)	
Midwest	0.97 (0.73–1.29)		1.14 (0.87–1.50)	
North	0.88 (0.64–1.21)		1.19 (0.86–1.65)	
South	0.75 (0.58–0.97)		0.73 (0.58–0.92)	
Population		< 0.001		< 0.001
≤ 20,000	1		1	
20,001–50,000	1.51 (1.03–2.22)		1.35 (0.91–1.97)	
50,001–150,000	2.37 (1.64–3.41)		1.88 (1.29–2.73)	
> 150,000	4.16 (2.93–5.91)		2.84 (1.94–4.14)	
HDI		< 0.001		< 0.001
Low (< 0,55)	1		1	
Average (0,55–0,699)	1.88 (0.64–5.55)		1.56 (0.53–4.52)	
High (0,70–0,799)	4.19 (1.44–12.21)		2.20 (0.73–6.63)	
Very high (≥ 0.80)	8.20 (2.81–23.84)		3.34 (1.09–10.24)	
Coverage of PHC		0.053		-
Absent	1			
Incipient	1.40 (0.71–2.76)		-	
Intermediate	1.18 (0.61–2.29)			
Consolidated	0.61 (0.32–1.18)			
Coverage of PHC (with oral health team)		< 0.001		-
Absent	1			
Incipient	1.42 (1.00–2.05)		-	
Intermediate	0.95 (0.65–1.39)			
Consolidated	0.60 (0.41–0.88)			
Average municipal wage (tertile)		< 0.001		-
1^st^ (low)	1			
2^nd^	1.62 (1.26–2.08)		-	
3^rd^ (high)	2.69 (2.16–3.36)			
Schooling rates		0.046		0.047
1^st^ (low)	1		1	
2^nd^	1,16 (0.93–1.44)		0.99 (0.80–1.23)	
3^rd^ (high)	1.30 (1.06–1.61)		1.23 (1.00–1.50)	
Infant mortality rate		< 0.001		-
1^st^ (low)	1			
2^nd^	0.98 (0.81–1.18)		-	
3^rd^ (high)	0.65 (0.52–0.82)			

-2 log-likelihood empty model = 659.13611; -2 log-likelihood adjusted model = 604.62142

**Figure 2:**
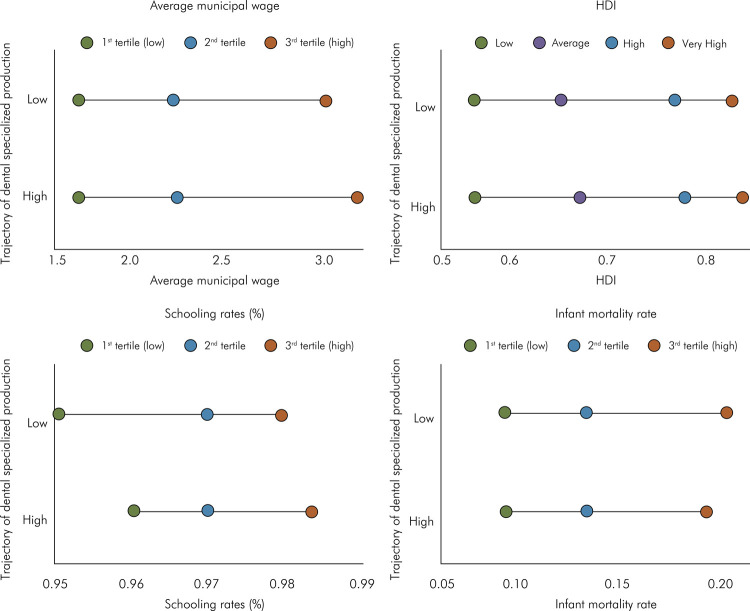
Distribution of average monthly salary, HDI, schooling rate, and infant mortality rate according to the trajectory of dental specialized production, Brazil. (n = 893 municipalities)

Figure 2 illustrates the inequalities of dental specialized production according to the contextual variables. Considering the analysis of absolute and relative inequalities, the positive results of the slope index showed the frequency of the high trajectory of dental specialized production was higher in the municipalities with the highest average monthly salary, with the best HDI, the lowest infant mortality rate, and the highest schooling rate. Thus, absolute inequality was 52.1% for the average monthly salary, 61.0% for the HDI, -22.1% for the infant mortality rate, and 14.8% for the schooling rate.

Similarly, the positive results of the concentration index indicating the frequency of a high trajectory in the achievement of goals was higher in the municipalities with higher average salary tertiles, with higher HDI, and lower mortality rates (Table 5).

**Table 5:** Absolute and relative inequalities in the trajectory of dental specialized production according to contextual factors, Brazil (n = 893 municipalities).

Variable	Trajectory of dental specialized production			
	SII (95%CI)	p-value	CIX (95%CI)	p-value
Average municipal wage	52.1 (42.8–61.3)	< 0.001	20.3 (15.4–25.2)	< 0.001
HDI	61.0 (52.5–69.3)	< 0.001	22.5 (17.8–27.2)	< 0.001
Infant mortality rate	-22.1 (-33.4–10.8)	< 0.001	- 10.5 (-15.2–-5.8)	< 0.001
Schooling rates	14.8 (3.3–26.2)	0.012	2.0 (-2.4–7.4)	0.320

SII: slope index; CIX: concentration index; CI95%: 95% confidence interval.

## Discussion

The present study demonstrates that most municipalities had a low trajectory of dental specialized procedures. There are marked inequalities in the trajectory of specialized dental care in Brazil, and absolute and relative inequalities were observed according to the local average monthly salary, HDI, and infant mortality rate. Furthermore, we observed that the local population size, HDI, the schooling rate, and the Brazilian region were the contextual variables that best predicted the trajectory of the dental specialized services, pointing out the effect of socioeconomic inequalities on dental health services.

The two most populated Brazilian regions (the Northeast and Southeast, respectively) had the highest number of registered and active DSCs during the evaluated period. Pedrazzi ^26^ and Goes ^27^ found a similar rate for the distribution of DSCs according to regions, revealing the maintenance of this indicator over time. Also, 62.9% of the municipalities had a low trajectory of dental specialized production; municipalities located in the southern regions had the lowest rates. Important absolute and relative inequalities in the trajectory were also observed. Municipalities with the best performance in dental specialized production were those with the best social indicators, especially HDI. Indeed, higher HDI, lower population density, and health facilities implemented within a shorter timeframe have shown better indicators of public services.^27,28^ These findings underscore the importance of public policies that adopt a broader perspective, fostering the encouragement and development of diverse social segments,^10,29^ considering that the National Oral Health Policy alone can not solve the mismatches in the assistance model.^30^

The organization and provision of health services seem to be related to human development in the major regions.^31^ However, Fernandes^32^ observed a pro-equity trend in the opportunity for accessing primary care in oral health instates with the lowest HDI. This could potentially lead to a decrease in oral health inequalities to some extent as a result of the implementation of current political measures. It is important to emphasize that more vulnerable populations do not have access to health care and, when they do, they end up receiving curative or iatrogenic treatment.^33^

The improvement in social indicators does not necessarily imply the reduction of oral health inequalities;^32^ however, states and municipalities with an improved HDI have shown better organization and greater provision of health services.^32^ Such information is valuable considering the formulation of public health policies. The present study demonstrated that municipalities with wide PHC oral health coverage possess the worst performance trajectories in the outpatient production of required secondary care, as previously observed.^28^ This finding highlights a possible mismatch between primary and secondary care in Brazil. We can speculate that public oral health policies may have guided, to some extent, the organization and expansion of primary care networks in more vulnerable regions, due to their low cost and high problem-solving ability. As a result of increased investment and greater availability of specialists, oral health services in secondary care have been structured into larger and better-organized centers. In line with these findings,^32^ an imbalance between specialized procedures and individual dental care was observed in states with higher and lower HDI, suggesting that incentives directed towards primary care for the most vulnerable regions (as programmatic targets) may have potentiated this effect.^5,10,32^

One of the most significant aspects of the present study is that it investigates factors associated with the trajectory of dental specialized production throughout the Brazilian territory, highlighting absolute and relative inequalities. The importance of the evaluations of the services offered by SUS is also highlighted for the improvement of health actions. The utilization of time series analysis in this investigation allows controlling for confounding, ensuring the study’s originality and reinforcing its importance. However, there are limitations in the present study associated with the use of secondary data, which may lead to underreporting or overreporting. Nevertheless, it can be assumed that these biases might have occurred in a non-systematic way across municipalities, thus decreasing their impact on the presented results. The findings described in this study can serve as support for the formulation of public health policies, as well as a tool for the integration and improvement of dentistry within the SUS framework.

## Conclusion

Most of the dental specialty centers analyzed herein showed a low trajectory of dental specialized production. Therefore, we emphasize the need to adjust the criteria and guidelines for the implementation and monitoring of secondary oral health care, devising more streamlined models for dental specialty centers that are flexible yet tailored to the epidemiological needs of the local population. We also observed marked inequities in the trajectory of dental specialized production based on contextual factors (local average monthly salary, HDI, and infant mortality rate). Thus, contextual characteristics at the local level, such as smaller city size, lower HDI, and lower schooling rate, lead to worse trajectories of dental specialized production.
